# Response to subsequent antiseizure medications after first antiseizure medication failure in newly diagnosed epilepsy

**DOI:** 10.3389/fneur.2022.1042168

**Published:** 2022-11-10

**Authors:** Hire Hersi, Jukka T. Saarinen, Jani Raitanen, Jukka Peltola

**Affiliations:** ^1^Department of Neurology, Vaasa Central Hospital, Vaasa, Finland; ^2^Faculty of Social Sciences (Health Sciences), Tampere University and the UKK Institute for Health Promotion Research, Tampere, Finland; ^3^Department of Neurology, Tampere University and Tampere University Hospital, Tampere, Finland

**Keywords:** seizure freedom after first antiseizure medication, drug-resistant epilepsy, ILAE classification, oxcarbazepine, valproic acid, carbamazepine

## Abstract

**Objective:**

There is a lack of studies using the International League Against Epilepsy (ILAE) recommendation to define drug-resistant epilepsy (DRE). This study evaluated the seizure freedom rates of substitution or add-on and subsequent antiseizure medication (ASM) therapies using different proposed definitions of DRE or ASM trials in patients with a failed first ASM. We also identified prognostic factors for 1-year seizure freedom.

**Methods:**

This study included 459 patients with epilepsy of whom 151 were not seizure-free after the first ASM. Multilevel mixed-effects logistic regression was used to examine the correlation between observations from the same patient.

**Results:**

The overall seizure freedom rate with the first and subsequent ASMs was 88.0% (404/459). The rate of DRE when defined as the failure of two ASMs for any reason was 20.0%, and according to the ILAE definition of DRE, it was 16.3%. After failing the first ASM, 63.6% of patients (96/151) became seizure free with subsequent ASMs and tried an average of 1.9 ASMs (range 1–5). Of the patients who achieved 1-year seizure freedom, 10.1% (41/404) were taking polytherapy and there was no difference between substitution and add-on. All the patients with generalized epilepsy were seizure-free. A favorable prognostic factor was age >60 years and an EEG without epileptiform activity. The efficacies of the different ASMs were largely similar, but drugs that enhanced GABA-mediated inhibitory neurotransmission had the lowest seizure freedom rate.

**Significance:**

In adults with newly-diagnosed epilepsy, 1-year seizure freedom was achieved for almost 90% of the patients. After failing the first ASM, two-thirds of the patients responded to subsequent ASM regimens. Our results support the feasibility and applicability of the ILAE concept of an adequate ASM trial and the failure of two ASMs as a definition of DRE.

## Key points

• The seizure freedom rate with the first and subsequent antiseizure medications was 88.0% (404/459). Therefore, 12.0% of the patients had absolute drug-resistant epilepsy.

• When the International League Against Epilepsy (ILAE) criteria for drug-resistant epilepsy–failure of two ASMs due to the lack of efficacy–was applied, 16.3% had drug-resistant epilepsy.

• Most patients (57.3%) who became seizure-free after failing their first antiseizure medication received monotherapy.

• Elderly patients (> 60 years old) were more likely to become seizure-free than patients aged 25–60 years (odds ratio = 2.75, *p* = 0.014).

• ASMs that enhanced GABA-mediated inhibitory neurotransmission had the lowest seizure freedom rate (14.3%).

## Introduction

Multiple factors influence the probability of seizure freedom in patients with newly diagnosed epilepsy, including the patient population, antiseizure medication (ASM) availability, and classification applied for the diagnostic criteria of epilepsy, seizure type, and epilepsy type. A landmark study of previously untreated patients with epilepsy found that 47% and 14% became seizure-free during treatment with their first and second, or third ASM, respectively. Eventually, 63% achieved at least 1-year of seizure freedom (1). Most previous studies used the total number of failed ASMs as a marker of refractoriness; however, there is a clear difference in the probability of achieving seizure freedom, depending on the reason for the discontinuation of a given ASM. In a randomized controlled trial, 70% of patients achieved 12-months remission, with a first treatment failure in 65% and 80% due to inadequate seizure control and side effects, respectively (2).

The International League Against Epilepsy (ILAE) provided a standardized definition in 2010 to enhance uniformity across studies and defined drug-resistant epilepsy (DRE) as the failure of adequate trials of two tolerated, appropriately chosen, and used ASM schedules, as a monotherapy or in combination, to achieve sustained seizure freedom (3). However, epidemiological studies applying this official recommendation are lacking. Some studies suggested the concept of absolute DRE that requires the failure of six ASMs because a significant minority of patients were rendered seizure-free with the addition of newly administered ASMs after the failure of two to five past ASMs (4). A hypothesis for differentiation between DRE and uncontrolled epilepsy was proposed because some patients had inadequate use of ASMs (5).

Prognostic factors for seizure freedom in patients with epilepsy and first ASM have been extensively studied (6). We have recently reported in a group of patients with newly diagnosed epilepsy that those with focal epilepsy with unknown etiology, normal electroencephalogram (EEG), or focal to bilateral tonic-clonic seizures (FBTCS) as the presenting seizure type had a better chance of obtaining seizure freedom with the first ASM than patients with structural or infectious etiology, epileptiform activity on EEG, or focal impaired awareness seizures (FIAS) (7). However, prognostic factors for achieving remission with the second or subsequent ASM regimens have been less explored. According to a recent study, seizure freedom with the second ASM was more probable in men and patients >45 years, and patients with generalized TCS or FBTCS before initiation of the first ASM were more likely to respond to the second ASM (2).

Although the advantages of ASM monotherapy in the initial management of epilepsy are widely accepted, there is no global agreement on treatment strategies when seizures continue after the initial monotherapy. Two different strategies with similar outcomes according to some studies have been used, the substitution of the initially ineffective ASM with another ASM administered as monotherapy or the administration of a second ASM as an add-on polytherapy (8, 9). In contrast, a recent study of patients in whom the first monotherapy failed due to the lack of efficacy reported that 51.0% of patients following substitution and 38.1% of patients with add-on achieved seizure remission (10). The role of combination therapy as a treatment strategy for epilepsy is being re-evaluated. Based on the drugs' perceived primary mechanism of action (MOA), it has been suggested that more patients become seizure-free when the combination involves a sodium channel blocker and a drug with multiple MOA compared with other combinations (11).

This study evaluated seizure freedom rates after failing the first ASM using different proposed definitions of the DRE and ASM trials. We also determined prognostic factors for seizure freedom, including the effect of second substitution or add-on ASM therapy and subsequent ASM therapies with different MOA combinations in patients who did not become seizure-free with the first ASM.

## Materials and methods

Originally, the study included 584 patients with epilepsy aged ≥16 years who were referred to the Tampere University Hospital (Pirkanmaa region, Finland) between January 1, 1995, and December 31, 2005. All individuals were retrospectively followed-up until at least 1-year of seizure freedom, December 31, 2006, or until death. Medical records were examined retrospectively, and after thorough validation of epilepsy diagnosis, 459 patients were finally included (7). Patients with alcohol and recreational drug abuse were excluded from our study because the seizures in these patients were considered provoked. According to the Finnish healthcare system, most newly diagnosed patients with epilepsy and practically all patients who continue to have seizures after the first ASM failure are treated within a public specialist service system. When adult patients reach 1 year of seizure freedom, their care is usually transferred to the general practitioner level, and if these patients have seizure relapses, their care is transferred back to a specialist clinic. Patients who continue to experience seizures continue to receive care at the specialist level.

ASM therapy was initiated according to the standard clinical practice during that period. If seizure freedom was not achieved with the past ASM, substitution or add-on ASMs were initiated at the treating physician's discretion, which reflects decision-making in a real-world context. Adequate ASM trials were identified using the criteria provided by the ILAE definitions (3).

Baseline characteristics were described as medians with interquartile ranges (IQRs) or frequencies with percentages. Depending on the variable, group comparisons were performed using Pearson's Chi-square test, the Mann–Whitney U test, or Fisher's exact test. Binary logistic regression was used to examine the association between seizure freedom following a second or subsequent ASM and sex. The Holm–Bonferroni method was used for multiple tests. We selected covariates based on the findings from our first analysis (7). Age at diagnosis (continuous), etiology (structural as a reference group), and seizure type (FBTCS as a reference group) were examined as potential confounding factors. Odds ratios (ORs) and 95% confidence intervals (CIs) were calculated for each covariate. We also examined the association between seizure freedom and ASM or ASM combinations. The same patient may have received two or more ASMs or ASM combinations; therefore, we used a multilevel mixed-effects logistic regression adjusted for the ASM regimen number to consider the correlation between observations from the same patient. A group of sodium channel blockers was used as the reference group, and ORs with 95% CIs were reported for other ASMs or ASM combinations categorized by their putative primary MOA. The data were analyzed using the software Stata version 16.1 (College Station, TX, United States). There was no contact with patients, and information was collected from the patient register of the Tampere University Hospital. This study does not require ethics committee approval according to Finnish Law on Research. Following Finnish guidelines, this study was approved by the head of the Tampere University Science Center.

## Results

### Seizure freedom rates according to different definitions of DRE

The baseline characteristics of all 459 previously untreated patients with validated epilepsy diagnoses were presented in detail in our previous study (7). The responses to the first and subsequent ASM schedules in absolute numbers using the ILAE definition of an adequate ASM trial are presented in Table 1. A total of 308 patients (76.2% of all patients achieving seizure freedom with ASM) became seizure-free following the administration of the first ASM. Therefore, 151 patients who continued to have seizures constituted the present study group. When using the ILAE definition of an adequate ASM trial, 346 patients (85.2% of all patients achieving seizure freedom) became seizure-free after the first ASM regimen.

**Table 1:** Antiseizure medication schedules.

	**Seizure freedom**					
	**# ASM Regimen**	**Total patients using these ASMs (*n*)**	**Total (*n*)**	**% of patients achieving seizure freedom with ASM**	**% of the total achieving seizure freedom (*n* = 406)**	**% of the total study cohort** **(*n* = 459)**
All patients regardless of the reasons for the initiation of subsequent antiseizure medication	1	459	308	67.1	75.9	67.1
	2	151	59	39.1	14.5	12.9
	3	66	22	33.3	5.4	4.8
	4	30	9	30.0	2.2	2.0
	5	10	4	40.0	1.0	0.87
	6	6	2	33.3	0.5	0.44
	Total	459	406*	na	99.5	88.0
Patients who used subsequent antiseizure medication only due to lack of efficacy	1	459	346	75.4	85.2	75.4
	2	102	38	37.3	9.4	8.3
	3	40	11	27.5	2.7	2.4
	4	18	6	33.3	1.5	1.3
	5	5	3	60.0	0.7	0.65
	6	2	0	-	-	-
	Total	459	406*	na	99.5	88.0

ASM, antiseizure medication; na, not applicable; n, number; ^*^, including two patients who became seizure free with epilepsy surgery.

**Figure 1:**
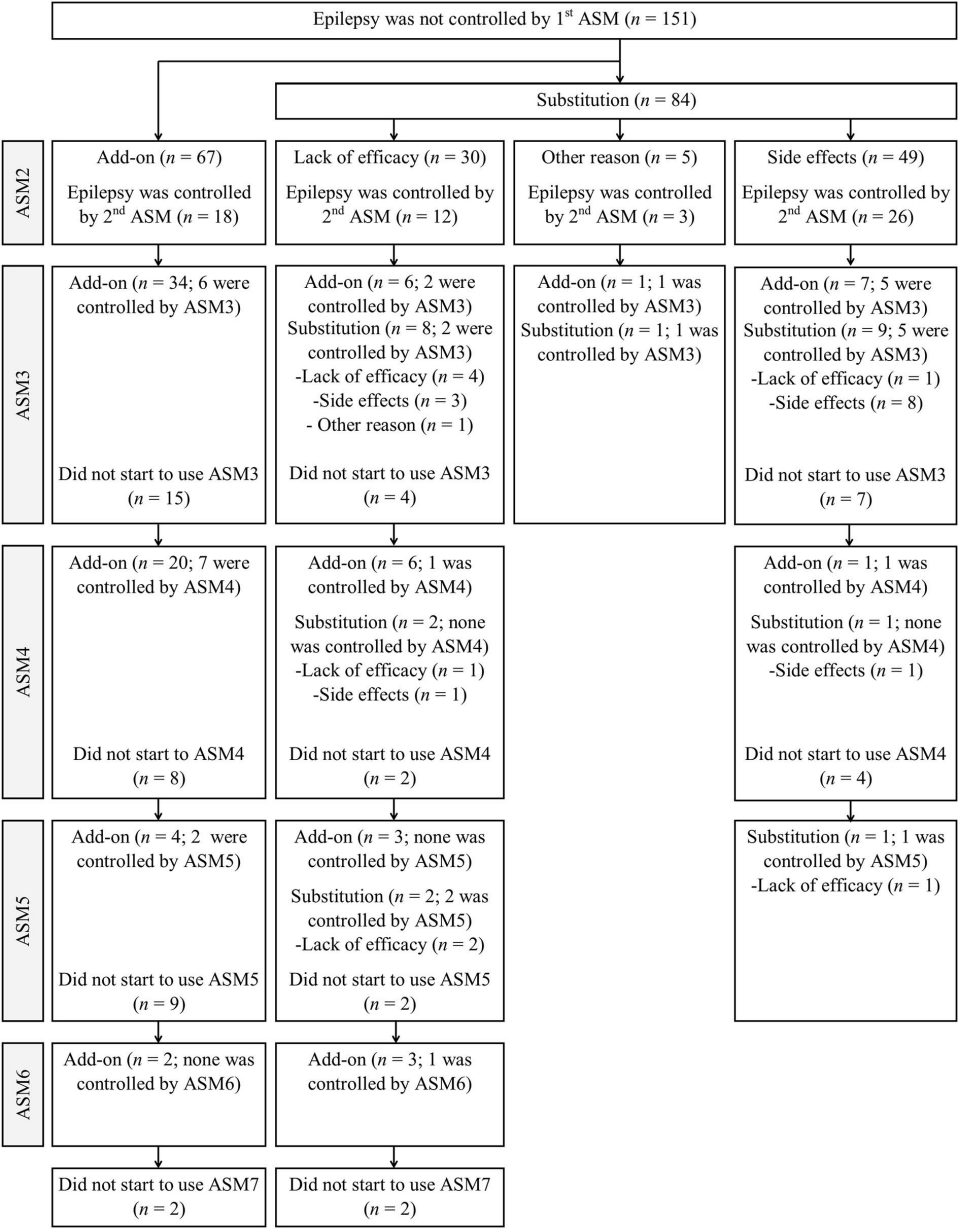
Patient responses to different combinations of the add-on and substitution ASMs after seizure freedom.

Fifty-nine of 151 patients (14.5% of all patients achieving seizure freedom) became seizure-free following the administration of the second overall ASM, and 38 of 102 patients became seizure-free with the second ASM regimen (9.4% of all patients achieving seizure freedom), according to the ILAE definition of an adequate trial (subsequent ASM was initiated only due to the lack of efficacy). Thirty-seven patients became seizure-free after the third to sixth ASM regimens when all ASM trials were included, compared with 20 patients who became seizure-free when the adequate ASM trial definition was used (5.4% third, 2.2% fourth, and 1.0% fifth ASM regimens vs. 2.7, 1.5, and 0.7% of all seizure-free patients, respectively). A minority of patients (28.3%, 26/92) did not start the third ASM due to shortness of follow-up or other reasons (Figure 1). Four patients had persistent seizures, even after six ASM trials. Two patients who had not become seizure-free with at least three ASMs underwent epilepsy surgery and subsequently became seizure-free.

The seizure freedom rate after the initiation of a second or subsequent ASM therapy in absolute numbers of patients in whom the first ASM treatment failed to control seizures was 63.6% (96/151) and 56.9% (58/102), according to the ILAE adequate trial definition. The seizure freedom rate with the first and subsequent ASMs was 88.0% (404/459). Therefore, 12.0% of the patients had an absolute DRE (six or more regimens were tried). The cumulative seizure freedom rate was 80.0% (367/459) after the second total ASM regimen and 83.7% (384/459) after two adequate trials, regardless of the reason for substitution or add-on. This indicates that 16.3% of the entire study population fulfilled the ILAE criteria for DRE. Conversely, in 20.0% of the patients, two ASMs failed to control seizures in absolute numbers.

### Prognostic factors for achieving seizure freedom either after the second ASM or after fulfilling the criteria for DRE (third or subsequent ASM regimen)

The clinical characteristics of all 151 (32.9%) patients who did not become seizure-free following the first ASM with reference to achieving seizure freedom either after the second ASM or after fulfilling the criteria for DRE (third or subsequent ASM regimens) are presented in Table 2. All seven patients with generalized epilepsy failing the first ASM became seizure-free following the second or subsequent ASM. Patients who became seizure-free with the second or subsequent ASM regimens were found to be significantly associated with the presenting seizure type and EEG. The likelihood of having FBTCS or FAS as the presenting seizure type and EEG without epileptiform activity was higher in patients who became seizure-free with the second ASM regimen, and they were also significantly older than patients who became seizure-free with the third or subsequent ASM regimens. Patients with persistent seizures were more likely to have epileptiform activity on EEG than those responding to the second ASM regimen. The follow-up time for patients with either persistent seizures or who had become seizure-free after the third or later ASM was significantly longer compared with those responding to the second ASM (6.0 years, 4.7 years, and 2.6 years, respectively).

**Table 2:** Background characteristics (median and interquartile range or frequency and percentage) at the last clinic visit for all patients with epilepsy who did not become seizure-free following administration of the first antiseizure medication.

	**All patients**	**Seizure freedom**		**Persistent seizures**	***p^1^***	***p* ^2^**
		**After 2^nd^ ASM**	**After 3^rd^ or later ASM**			
*N*	151	59	39	53		
Sex, *n* (%)					0.178^3^	0.992^3^
Female	83 (55.0)	30 (50.8)	26 (66.7)	27 (50.9)		
Male	68 (45.0)	29 (49.2)	13 (33.3)	26 (49.1)		
Duration of follow-up, med (IQR)	4.2 (2.5–6.9)	2.6 (1.4–4.6)	4.7 (2.9–7.0)	6.0 (4.2–9.0)	0.001^4*^	<0.001^4*^
Age at diagnosis, med (IQR)	44 (27–59)	51 (35–70)	28 (21–53)	42 (31–53)	0.007^4*^	0.088^4^
Epilepsy type, *n* (%)					0.063^3^	0.497^5^
Focal	144 (95.4)	57 (96.6)	34 (87.2)	53 (100)		
Generalized	7 (4.6)	2 (3.4)	5 (12.8)	0		
Etiology, *n* (%)					0.161^3^	0.224^5^
Structural	94 (62.3)	35 (59.3)	22 (56.4)	37 (69.8)		
Genetic	7 (4.6)	2 (3.4)	5 (12.8)	0		
Infectious	6 (4.0)	1 (1.7)	2 (5.1)	3 (5.7)		
Unknown	44 (29.1)	21 (35.6)	10 (25.6)	13 (24.5)		
Type of 1^st^ seizure, *n* (%)					0.021^3*^	0.095^5^
FBTCS	98 (64.9)	42 (71.2)	22 (56.4)	34 (64.2)		
FAS	25 (16.6)	12 (20.3)	4 (10.2)	9 (17.0)		
FIAS	21 (13.9)	3 (5.1)	8 (20.5)	10 (18.9)		
GTCS	3 (2.0)	1 (1.7)	2 (5.1)	0		
Myoclonic	4 (2.6)	1 (1.7)	3 (7.7)	0		
EEG, *n* (%)					0.004^3*^	0.011^3*^
Normal	51 (33.8)	28 (47.5)	9 (23.1)	14 (26.4)		
Epileptiform activity	42 (27.8)	9 (15.3)	17 (43.6)	16 (30.2)		
Focal slowing	20 (13.2)	7 (11.9)	5 (12.8)	8 (15.1)		
Unspecific	18 (11.9)	3 (5.1)	5 (12.8)	10 (18.9)		
No EEG	20 (13.2)	12 (20.3)	3 (7.7)	5 (9.4)		

^1^, p-value for comparison between seizure freedom after the 3^rd^ or later ASM (two patients who became seizure-free after epilepsy surgery are not included) and seizure freedom after the 2^nd^ ASM.

^2^, p-value for comparison between persistent seizures and seizure freedom after the 2^nd^ ASM.

^3^, Chi-squared test.

^4^, Mann-Whitney U test.

^5^, Fisher's exact test.

^*^Denotes statistically significant association using the Holm-Bonferroni correction (thresholds for the lower and higher p-value are 0.025 and 0.05).

ASM, antiseizure medication; FBTCS, focal to bilateral tonic-clonic seizures; FAS, focal aware seizures; FIAS, focal impaired.

awareness seizures; GTCS, generalized tonic-clonic seizures, IQR, interquartile range, med, median.

At the time of diagnosis, most patients (86/151, 57.0%) were 25–60 years of age, whereas 19.9% (30/151) had epilepsy diagnosed between 16–25 years, and 23.2% (35 of 151) were elderly (>60 years old). The cumulative seizure freedom rate for focal epilepsy was 85.7% (12/14) in patients aged 16–25 years, 50.6% (43/85) in patients aged 25–60 years, and 75.6% (34/45) in elderly patients, who were more likely to become seizure-free than those aged 25–60 years (OR = 2.75, *p* = 0.014). There was no difference in cumulative seizure freedom between young (18–25 years of age) and elderly patients (OR = 0.52, *p* = 0.429). Among women of childbearing age (ages 16–46 years), 25.0% (11/44) had valproic acid (VPA) as a second or subsequent ASM. Eight of these patients had focal epilepsy and three had generalized epilepsy. The seizure-freedom rate was 81.8% (9/11).

With regard to prognostic factors for seizure freedom in patients with focal epilepsy, detailed information about the association between sex, age at diagnosis, type of first seizure, etiology, and EEG is presented in Table 3. Patients with epilepsy due to an unknown reason had a trend for higher odds (OR = 2.05, 95% CI: 0.84–5.01) of seizure freedom than patients with structural etiology. Patients with FIAS as their presenting seizure were less likely to achieve seizure freedom than those with FBTCS but this trend was not significant. The seizure freedom rate with a second or subsequent ASM in focal epilepsy was 61.8% (89/144), with no significant differences related to sex, etiology, type of the first seizure, or EEG. The seizure freedom rate for focal epilepsy was 62.3% in females and 61.2% in males (*p* = 0.888). With structural and unknown etiologies, seizure freedom rates were 59.6 and 68.2%, respectively. The seizure freedom rates for the FBTCS, FAS, and FIAS as the presenting seizure types were 65.3, 64.0, and 52.4%, respectively.

**Table 3:** Odds ratios and 95% confidence intervals and *p* values from the logistic regression models for seizure freedom after second or subsequent antiseizure medications in patients with focal epilepsy.

	**Model 1**		**Model 2**	
	**OR (95% CI)**	***p***	**OR (95% CI)**	***p***
Sex (ref. = female)	0.95 (0.47–1.94)	0.898	0.81 (0.38–1.73)	0.594
Age at date of diagnosis	1.02 (1.00–1.04)	0.123	1.02 (0.99–1.04)	0.229
**Type of 1**^**st**^ **seizure (ref**. **=** **FBTCS)**	
FAS	0.96 (0.38–2.44)	0.934	0.76 (0.28–2.01)	0.575
FIAS	0.63 (0.24–1.67)	0.352	0.64 (0.23–1.74)	0.382
**Etiology (ref**. **=** **structural)**	
Infectious	0.88 (0.16–4.90)	0.882	0.83 (0.14–4.79)	0.835
Unknown	2.05 (0.84–5.01)	0.114	1.72 (0.66–4.43)	0.264
**EEG (ref**. **=** **normal)**	
Epileptiform activity			0.60 (0.23–1.53)	0.283
Focal slowing			0.57 (0.17–1.91)	0.359
Unspecific activity			0.34 (0.10–1.14)	0.080
No EEG			1.05 (0.28–3.86)	0.944

CI, confidence interval; FAS, focal aware seizures; FBTCS, focal to bilateral tonic-clonic seizures; FIAS, focal impaired awareness seizures; OR, odds ratio; ref, reference group.

### Monotherapy vs. polytherapy after the first ASM failure

The differences in background characteristics between the add-on and substitution subgroups due to the lack of efficacy are shown in Table 4. Additionally, more details on patient responses to different combinations of the add-on and substitution ASMs after seizure freedom was not achieved with the administration of the first ASM are presented in Figure 1. Patients who became seizure-free after failing the first ASM had an average of 1.9 ASMs (standard deviation, 1.0; range: 1–5). Most patients (57.3%, 55/96) received monotherapy and two ASMs were used concurrently by 39.6% (38/96) of the patients. Only two patients (2.1%) used three ASMs simultaneously and one patient (1.0%) used four ASMs simultaneously. Among the patients who achieved 1-year seizure freedom in the entire cohort, 10.1% (41/404) were on combination therapy.

**Table 4:** Baseline characteristics of the patients when the first ASM was substituted or another ASM was combined (add-on) because of lack of efficacy.

	**Add-on**	**Substitution**	***p***
*N*	52	50	
**Sex**, ***n*** **(%)**			0.698^1^
Female	24 (46.2)	25 (50.0)	
Male	28 (52.8)	25 (50.0)	
Duration of follow-up, med (IQR)	4.6 (3.2–7.6)	4.5 (2.6–6.5)	0.357^2^
Age at diagnosis, med (IQR)	32.5 (21–52)	49.0 (28–55)	0.074^2^
**Epilepsy type**, ***n*** **(%)**	
Focal	49 (94.2)	47 (94.0)	0.961^1^
Generalized	3 (5.8)	3 (6.0)	
Etiology, *n* (%)			0.540^1^
Structural	39 (57.7)	35 (70.0)	
Genetic	3 (5.8)	3 (6.0)	
Infectious	2 (3.8)	2 (4.0)	
Unknown	17 (32.7)	10 (20.0)	
Type of 1^st^ seizure, *n* (%)			0.519^1^
FBTCS	30 (57.7)	33 (66.0)	
FAS	7 (13.5)	9 (18.0)	
FIAS	12 (23.1)	5 (10.0)	
GTCS	1 (1.9)	1 (2.0)	
Myoclonic	2 (3.8)	2 (4.0)	
**EEG**			0.734^1^
Normal	18 (34.6)	16 (32.0)	
Epileptiform activity	16 (30.8)	14 (28.0)	
Focal slowing	5 (9.6)	8 (16.0)	
Unspecific activity	6 (11.5)	8 (16.0)	
No EEG	7 (13.5)	4 (8.0)	

^1^Chi-squared test; ^2^Mann–Whitney U test.

FBTCS, focal to bilateral tonic-clonic seizures; FAS, focal aware seizures; FIAS, focal impaired awareness seizures; GTCS, generalized tonic-clonic seizures; IQR, interquartile range; med, median.

The seizure freedom rates were 53.0% (26/49) and 40.0% (12/30) in the subgroup of first substitutions when the substitution was due to side effects and lack of efficacy, respectively. When the patient was given the first add-on ASM after seizure freedom was not achieved, 26.9% (18/67) became seizure-free. When the first ASM was substituted (*n* = 30) or another ASM was combined owing to the lack of efficacy after subsequent ASMs (*n* = 67), the final seizure freedom rate was 54.6% (53/97). When the first ASM was changed due to side effects or other reasons after subsequent ASMs, 79.6% (43/54) eventually became seizure-free.

The efficacy of individual ASMs when used in monotherapy and polytherapy was combined for the treatment of focal epilepsy was not significantly different compared with VPA when controlling the ASM regimen or combination number (Table 5). Carbamazepine (CBZ) had the highest seizure freedom rate (64.4%), followed by oxcarbazepine (OXC), phenytoin, and VPA (55.8, 55.2, and 54.7%, respectively).

**Table 5:** Efficacy of antiseizure medications used in mono- or polytherapy.

	**Seizure freedom**				
	**No, *n* (%)**	**Yes, *n* (%)**	**Total**	**OR (95% CI)**	***p***
Oxcarbazepine	168 (44.2)	212 (55.8)	380	0.83 (0.19–3.64)	0.809
Valproic acid	53 (45.3)	64 (54.7)	117	1.00 (reference group)	
Carbamazepine	32 (35.6)	58 (64.4)	90	0.83 (0.11–6.25)	0.853
Lamotrigine	52 (63.4)	30 (36.6)	82	1.19 (0.18–7.65)	0.856
Levetiracetam	23 (57.5)	17 (42.5)	40	1.17 (0.12–11.2)	0.889
Topiramate	26 (72.2)	10 (27.8)	36	0.57 (0.50–6.57)	0.656
Phenytoin	13 (44.8)	16 (55.2)	29	2.21 (0.13–38.1)	0.585
Gabapentin	16 (69.6)	7 (30.4)	23	0.58 (0.04–9.18)	0.700
Clobazam	15 (88.2)	2 (11.8)	17	0.14 (0.003–7.46)	0.330
Tiagabine	15 (93.8)	1 (6.2)	16	0.12 (0.004–33.6)	0.464
Clonazepam	9 (75.0)	3 (25.0)	12	0.30 (0.002–41.6)	0.635
Phenobarbital	3 (100)	0	3		
Benzodiazepine	1 (100)	0	1		
Pregabalin	1 (100)	0	1		

Odds ratios (ORs) and 95% confidence intervals (CIs) and *p* value for seizure freedom from multilevel mixed-effects logistic regression model adjusted for the regimen number.

The seizure freedom rates for the 15 most commonly used monotherapy or polytherapy ASM combinations (of the total 70 regimens) in focal epilepsy, using VPA monotherapy (70.4% seizure-free) as the reference group, are presented in Table 6. There was no significant difference in achieving seizure freedom in any of the monotherapy options compared with the reference group (VPA). The combinations consisting of OXC/VPA (14.3% seizure-free), OXC/gabapentin (23.1% seizure-free), and OXC/lamotrigine (LTG) (28.6%) had significantly lower odds for seizure freedom compared with VPA. The combination of VPA with LTG reached a seizure-free rate of 44.4%, which was the third highest among polytherapy combinations after LTG/LEV and OXC/LEV (57.1% and 50.0%, respectively).

**Table 6:** Different substitutions or add-on combinations of antiseizure medications were used at least five patients.

	**Seizure freedom**				
	**No, *n* (%)**	**Yes, *n* (%)**	**Total**	**OR (95% CI)**	***p***
OXC	107 (35.8)	192 (64.2)	299	0.59 (0.33–1.06)	0.079
VPA	24 (29.6)	57 (70.4)	81	1.00 (reference group)	
CBZ	24 (30.8)	54 (69.2)	78	0.71 (0.34–1.48)	0.365
LTG	14 (48.3)	15 (51.7)	29	0.52 (0.21–1.29)	0.158
PHT	9 (37.5)	15 (62.5)	24	0.72 (0.27–1.91)	0.512
LTG + OXC	10 (71.4)	4 (28.6)	14	0.26 (0.07–0.96)	0.044
GBP + OXC	10 (76.9)	3 (23.1)	13	0.18 (0.04–0.77)	0.020
LEV + OXC	6 (50.0)	6 (50.0)	12	0.43 (0.11–1.67)	0.223
OXC + TPM	7 (63.6)	4 (36.4)	11	0.32 (0.08–1.30)	0.113
LTG + VPA	5 (55.6)	4 (44.4)	9	0.57 (0.13–2.59)	0.471
LEV	5 (55.6)	4 (44.4)	9	0.42 (0.09–1.90)	0.259
TPM	5 (62.5)	3 (37.5)	8	0.32 (0.07–1.51)	0.150
LEV + LTG	3 (42.9)	4 (57.1)	7	0.66 (0.11–3.88)	0.644
OXC + VPA	6 (85.7)	1 (14.3)	7	0.09 (0.01–0.86)	0.036
GBP	2 (40.0)	3 (60.0)	5	0.88 (0.13–5.77)	0.891

Odds ratios (ORs) and 95% confidence intervals (CIs) and *p* values for seizure freedom from the multilevel logistic regression model adjusted for the order of medications.

CBZ, carbamazepine; GBP, gabapentin; OXC, oxcarbazepine; LEV, levetiracetam; LTG, lamotrigine; PHT, phenytoin; TPM, topiramate; VPA, valproic acid.

The efficacies of different ASM groups based on the ASM MOA in focal epilepsy are presented in Table 7. Antiseizure medications (ASMs) with enhanced gamma-aminobutyric acid (GABA)-mediated inhibitory neurotransmission was less effective compared with ASMs that modulated voltage-gated sodium channels (14.3% vs. 64.5%) but the finding was not significant when controlling the ASM regimen or combination number (OR = 0.04, *p* = 0.098). The combination of two ASMs, compared with one ASM that modulated voltage-gated sodium channels alone, was not effective. The results were similar even when levetiracetam was separated into this group. Patients >60 years of age used VPA more frequently than patients aged 25–60 years (37.7% vs. 14.6%).

**Table 7:** Efficacy of antiseizure medications by different groups based on mechanism of action.

	**Seizure freedom**			
**ASM group**	**No, *n* (%)**	**Yes, *n* (%)**	**OR (95% CI)**	***p***
1	152 (35.5)	276 (64.5)	1.00 (reference group)	
2	6 (85.7)	1 (14.3)	0.04 (0.001–1.78)	0.098
3	6 (46.2)	7 (53.9)	0.61 (0.09–4.33)	0.624
4	28 (31.8)	60 (68.2)	1.28 (0.49–3.35)	0.620
1 + 2	14 (87.5)	2 (12.5)	0.03 (0.001–1.21)	0.063
1 + 1	14 (70.0)	6 (30.0)	0.35 (0.06–1.90)	0.223
1 + 3	19 (59.4)	13 (40.6)	0.26 (0.03–2.14)	0.211
1 + 4	20 (66.7)	10 (33.3)	0.27 (0.04–2.00)	0.199
3 + 4	8 (88.9)	1 (11.1)	0.01 (0.00003–2.50)	0.101
2 + 2	1	0		
2 + 3	1	0		
4 + 4	1	0		
1 + 1 + 2	3 (75.0)	1 (25.0)		
1 + 1 + 3	2	0		
1 + 1 + 4	3	0		
1 + 2 + 3	2	0		
1 + 2 + 4	7	0		
1 + 3 + 3	1	0		
1 + 3 + 4	1	0		
1 + 4 + 4	1	0		
2 + 2 + 4	1	0		
2 + 3 + 4	1	0		
3 + 3 + 4	0	1		
1 + 1 + 2 + 4	1	0		
1 + 2 + 4 + 4	2	0		
2 + 3 + 4 + 4	0	1		

Odds ratios (ORs) and 95% confidence intervals (CIs) and *p* values for seizure freedom from the multilevel logistic regression model adjusted for the order of medications. ASM, antiseizure medication.

Group 1: modulation of voltage-gated sodium channels.

- phenytoin, carbamazepine, oxcarbazepine, lamotrigine.

Group 2: enhancement of GABA-mediated inhibitory neurotransmission.

- benzodiazepine, tiagabine, clobazam, clonazepam, phenobarbital.

Group 3: modulation of neurotransmitter release via a presynaptic action.

- levetiracetam, gabapentin, pregabalin.

Group 4: multiple mechanisms of action.

- valproic acid, topiramate.

Table 8 summarizes the reasons for the first, second, and subsequent ASM withdrawal in patients with focal epilepsy. A total of 73 and 78 ASMs were discontinued owing to lack of efficacy and side effects, respectively. Oxcarbazepine (OXC), CBZ, LTG, and VPA were discontinued because of side effects in 12.1% (37/307), 42.3% (11/26), 13.5% (7/52), and 13.8% (8/58) of patients, respectively. These differences were statistically significant (*p* = 0.004).

**Table 8:** Reasons for discontinuation in focal epilepsy based on the entire history of antiseizure medication.

	**Lack of efficacy**	**Side effects**	**Other reason**	**Total**
Oxcarbazepine	32	38	3	73
Carbamazepine	8	11	1	20
Valproic acid	9	8	2	19
Lamotrigine	4	7	0	11
Phenytoin	4	4	3	11
Topiramate	4	2	1	7
Tiagabine	5	1	1	7
Clobazam	1	4	2	7
Gabapentin	4	1	0	5
Levetiracetam	2	2	0	4

## Discussion

Our study provides new insights into the prognosis of newly diagnosed epilepsy and emphasizes the significance of different definitions of ASM trials and DRE. We provide evidence that the age of onset-related composition of the study group plays a major role in the probability of achieving seizure freedom. We also identified that factors other than age influenced seizure outcomes, including seizure type and EEG findings. Owing to the limitations in statistical power, many of these findings are trending. Our analyses of the selection of specific ASMs demonstrate the inherent difficulty in achieving significant findings in a real-world setting owing to a large number of available ASM choices.

The overall initial 1-year seizure freedom rate for all ASM regimens was 88.0%, which was higher than the 63.7% seizure freedom rate observed in previous studies (1,2). When the ILAE-defined ASM trial was used, the seizure freedom rate for the first ASM increased from 67.1 to 75.4% in the total study cohort and from 75.9 to 85.2% in patients who achieved seizure freedom with the use of subsequent ASMs. The use of an adequate ASM trial definition decreased the proportion of patients who achieved seizure freedom with the second ASM from 12.9 to 8.3% of the total study cohort and from 14.6 to 9.4% of patients who achieved seizure freedom with the use of ASMs. Taken together, 16.6% of the entire study population fulfilled the ILAE criteria for DRE, but in 20.0% of the patients, two ASMs failed to control the seizures in absolute numbers. The proportion of patients achieving seizure freedom following the administration of the third to fifth ASM regimens decreased from 2.7 to 0.74% with each subsequent ASM regimen, further validating the relevance of the ILAE definition of DRE (3).

Increasing the number of ASM regimen trials increased the likelihood of seizure freedom, but not all patients in whom two ASM regimens failed to stop seizures initiated further ASM regimens. Therefore, uncontrolled epilepsy is not equivalent to DRE. The most common reason for this is the inadequate use of prescribed ASM(s) (5). A Scottish study reported that 74.2% (742/1,000) of patients who did not achieve seizure freedom with the first ASM tried a second one (12). In our study, all patients tried the second ASM, and 71.7% (66/92) of the patients tried a subsequent ASM. A significant number of patients (40.2%) (37/92) were also rendered seizure-free with the addition of ASMs, even after the failure of two to five previous ASMs. This finding indicates a substantially higher seizure freedom rate than previously reported (4). Patients with a history of recreational drug use have a 64% reduced chance of achieving terminal seizure freedom (5). Patients with alcohol and recreational drug use were excluded from our study because the seizures in these patients were considered provoked. This exclusion may at least partly explain the high seizure-free rates in our study.

The age distribution of patients did have a significant effect on the total seizure-free outcomes in our study, which did not include a large patient population with the onset of epilepsy in infancy and childhood (<16 years of age) who might respond differently to ASMs. Previous studies reported that there was no difference in the rate of terminal remission between adults and children, but patients with epilepsy with the onset in their 20s had the lowest remission probability (13, 14). In a 30-year Scottish longitudinal cohort study, the median age at referral was 33 years compared with 45 years at the time of diagnosis in our study (7, 12). Multivariable analysis of patients aged >70 years in a previous study revealed an OR of 2.25 for 12-months remission after the first treatment failure (2). Elderly patients with focal epilepsy were also more likely to be seizure-free in our study. Moreover, in our study, the patients who became seizure-free with the second ASM regimen were significantly older (mean age 51 years) than those who were free with the third or subsequent regimens (mean age 32 years). Even patients with drug resistant poststroke epilepsy tended to be younger with a mean age of 52 years according to a recent study (15).

All patients with generalized epilepsy in our study became seizure-free, consistent with our previous study (7). Additionally, patients who became seizure-free with the second ASM regimen were more likely to have FBTCS or FAS as the presenting seizure type and to have EEG without epileptiform activity compared with those who became seizure-free with the third or subsequent regimens. In addition, patients with persistent seizures were significantly more likely to have epileptiform activity on EEG than those responding to the second ASM regimen. Both features were also significant for the possibility of seizure freedom with the first ASM (7). The follow-up time for patients with either persistent seizures or becoming seizure-free after the third or later ASMs was significantly longer compared with those responding to the second ASM (6.0 years, 4.7 years, and 2.6 years, respectively), which is explained by the treatment guidelines in Finland where patients are followed up in a specialist center until 1-year seizure freedom is reached.

We did not detect significant differences in seizure freedom related to sex or etiology, which may be due to the limited number of patients in our cohort.

It has been proposed that when the first ASM fails due to lack of efficacy, add-on therapy should be initiated immediately because it is more effective than its application after the second ASM failure, possibly due to the concept of seizures begetting seizures, that is, secondary epileptogenesis (9). However, our study found no differences in efficacy when add-on therapy was used after the first ASM failed. This finding may be explained by a bias from the treating physician, who may have chosen substitution for patients who were estimated to have a better prognosis, and add-on therapy was offered to patients who were thought to have a worse prognosis in achieving seizure freedom. This bias may explain why patients in the add-on strategy tended to be younger than those in the substitution strategy.

When analyzing the efficacy of different ASMs, the highest seizure freedom rate was achieved with CBZ (65.9%) either in monotherapy or polytherapy in focal epilepsy without significant difference compared with other ASMs, where seizure-freedom rates ranged from 11.8% (clobazam) to 55.8% (OXC); only tiagabine had a significantly lower seizure freedom rate (6.7%). The low proportion of FIAS in our cohort may also be due to the lack of recognition of these seizures (16). This result may also explain why VPA had favorable efficacy in our study because it had good efficacy in FBTCS but was suboptimal in FAS and FIAS compared with CBZ (17). The favorable efficacy of VPA likely reflects physicians' preference to initiate VPA in older patients who generally have better responses to ASM.

At the group level with regard to the MOA, in monotherapy, ASMs with multiple MOA or with modulation of voltage-gated sodium channels had the highest seizure freedom rates (67.4 and 64.5%, respectively) compared with ASMs modulating neurotransmitter release *via* a presynaptic action (53.9%) without a significant difference. Conversely, ASMs that enhanced GABA-mediated inhibitory neurotransmission had the lowest seizure freedom rate (14.3%; *p* = 0.098). This is in line with an earlier study reporting that none of the patients who received a combination of a sodium channel blocker and GABAergic agent became seizure-free (8).

Lack of efficacy (45%) and side effects (47%) were the most common reasons for discontinuation of the initial and subsequent ASMs. CBZ had the highest rate of discontinuation owing to side effects when used in monotherapy and polytherapy. Treatment with CBZ is associated with a higher risk of discontinuation than treatment with LTG, LEV, or VPA in elderly individuals (18).

Owing to the retrospective study design, selection bias is a potential limitation of the present study. A modest sample size reduced the power required to determine the effect of combined ASMs. We were unable to document the possible underreporting of seizures. Our cohort also consisted of patients from an era when newer ASMs were non–existent or not widely used. However, CBZ, OXC, and VPA are currently chosen as first-line ASMs for focal epilepsy in Finland owing to the reimbursement policy, and many newer ASMs are reimbursed only when used as an add-on therapy but not as a substitution. However, new ASMs have not improved the probability of seizure freedom (12). On the other hand, there is a paucity of studies that have been performed recently analyzing in more detail the efficacy of subsequent ASM regimens including more newer generation ASMs. Therefore, a new study with a similar approach to our study but from a more recent period would be much warranted. Especially there is preliminary evidence of higher seizure freedom rates with cenobamate compared with older drugs (19). A major contribution to timely referral for epilepsy surgery was based on the official ILAE definition of DRE as a failure of two appropriate drug trials introduced in early 2010 (3). Because of our study design, an initial seizure freedom rate of at least 1 year (time to first remission) was used; however, long-term seizure freedom rates were not available. The proportion of relapsing-remitting courses of epilepsy was estimated as 16–52% depending on the patient population (20). Owing to the reasonably long follow-up time, some patients may have become seizure-free due to the natural disease course, regardless of medication. Finally, we did not have information available about psychiatric comorbidities or the number of pre-treatment seizures limiting the analysis of all possible relevant factors.

One of the key issues about the present study is how well the results from our single center can be generalized to other regions and patient populations? First, we have only included patients from adult neurology department (i.e., patients aged 16 years or more); which also explains why there are so few patients with generalized epilepsy because in the majority of those patients the onset of epilepsy is <16 years. On the other hand, our center covers a well-defined geographical area and is practically population-based. Moreover, our patient population does not represent a typical DRE population, because in order to be included in the original study population the patients needed to be newly diagnosed and the development of DRE was one of the outcomes of the study.

Our study provides new data for the prediction of seizure freedom in the adult population, providing a more positive outlook than previous studies. The results of our study support the feasibility and applicability of the ILAE concept of an adequate ASM trial, with further emphasis on the prognostic significance of the first adequate ASM trial and the failure of two ASMs as a definition of DRE.

## Data availability statement

The raw data supporting the conclusions of this article will be made available by the authors, without undue reservation.

## Ethics statement

Ethical review and approval was not required for the study on human participants in accordance with the local legislation and institutional requirements. Written informed consent from the patients/participants or patients/participants' legal guardian/next of kin was not required to participate in this study in accordance with the national legislation and the institutional requirements'.

## Author contributions

JP and JS conceived and designed the study plan. JS and HH drafted the manuscript. JR performed the statistical analyses and aided in manuscript writing. All authors read and reviewed the manuscript and approved the final version.

## Conflict of interest

Author JP has participated in clinical trials for Eisai, UCB, and Bial; received research grants from Eisai, Medtronic, UCB, and Liva-Nova; received speaker honoraria from LivaNova, Eisai, Medtronic, Orion Pharma, and UCB; received support for travel to congresses from LivaNova, Eisai, Medtronic, and UCB; and participated in advisory boards for Arvelle, Novartis, LivaNova, Eisai, Medtronic, UCB, and Pfizer. The remaining authors declare that the research was conducted in the absence of any commercial or financial relationships that could be construed as a potential conflict of interest.

## Publisher's note

All claims expressed in this article are solely those of the authors and do not necessarily represent those of their affiliated organizations, or those of the publisher, the editors and the reviewers. Any product that may be evaluated in this article, or claim that may be made by its manufacturer, is not guaranteed or endorsed by the publisher.
